# Antiviral Defense in Invertebrates

**DOI:** 10.3390/v10080403

**Published:** 2018-07-31

**Authors:** Michelle L. Flenniken

**Affiliations:** 1 Department of Plant Sciences and Plant Pathology, Bozeman, MT 59717, USA; michelle.flenniken@montana.edu; Tel.: +1-406-994-7229; 2 Center for Pollinator Health, Montana State University, Bozeman, MT 59717, USA

Invertebrate organisms include vectors of human viruses (mosquitoes, sand flies), model organisms (fruit fly), insect pollinators (honey bees and bumble bees), plant virus vectors (aphids), and commercially valuable aquatic species (oysters and shrimp) that play important roles in shaping ecosystems throughout the world. Like all organisms, invertebrates are infected by viruses and have, in turn, evolved strategies to limit virus infection. There are some fundamental similarities in host defense mechanisms, including the host recognition of non-self, pathogen-associated molecular patterns (e.g., viral dsRNA) that in turn stimulate the activation of host proteins, and expression of genes required to restrict virus replication, as well as unique aspects of specific host–virus interactions that are a result of co-evolution. Invertebrate antiviral defense mechanisms include canonical immune signaling cascades (e.g., Jak/STAT, Toll, Imd), heat shock responses, apoptosis, and dsRNA-triggered responses including the sequence-specific RNA interference mechanism and a less well characterized, non-sequence-specific dsRNA mediated response.

This special issue of *Viruses* features articles written by prominent researchers in the field of invertebrate immunology. The goal of this compilation is to better understand the similarities and differences in antiviral responses mounted by invertebrates, while recognizing that some of what is learned is unique to specific virus–host pairs. Interesting parallels can be drawn between invertebrate and vertebrate antiviral responses and, in some cases, homologous genes that play similar roles in a wide range of organisms have been identified. Together the articles in this issue serve as a foundation and inspiration for future studies aimed at further elucidating the molecular mechanisms of invertebrate antiviral defense.

Review articles led by Green and Speck, van Rij, Smagghe, Flenniken, Newton and Hardy, summarize the current understanding of antiviral responses in Pacific oysters (*Crassostrea giga*), fruit flies (*Drosophila melanogaster*), mosquito species (*Aedes* spp., *Culex* spp., *Anopholes* spp.), honey bees (*Apis mellifera*), and bumblebees (*Bombus* spp.), as well as describe the influence of endosymbionts on virus replication in fruit flies and mosquitos at the cellular level [1,2,3,4,5]. Antiviral defense in these organisms involves both non-specific innate immune responses including the interferon-like antiviral response mounted by Pacific oysters treated with a synthetic mimic of dsRNA, poly(I:C), which limits virus (i.e., Ostreid herpesvirus I) replication within treated oysters, as well as in the next generation [1]. Similarly, a non-sequence-specific dsRNA-mediated virus-limiting response is important in shrimp, honey bees, bumble bees, and sand flies, but less so in fruit flies and mosquitos [1,2,3,4,6]. Although Dicer-mediated transcriptional activation of antiviral genes, including *vago*, in response to dsRNA is important in fruit flies and mosquitos, the predominant dsRNA-triggered antiviral mechanism is RNA interference (RNAi), which is a sequence-specific post-transcriptional silencing mechanism [2,3]. RNAi is also involved in limiting virus infections in honey bees and bumble bees, but the relative importance of this and other mechanisms for particular virus infections remains to be elucidated. Furthermore, it is interesting to speculate that the differences in responses may partially be attributed to different evolutionary pressures in social versus solitary insects [4]. One of the most well-studied bee host—virus interactions is deformed wing virus (DWV) infection of honey bees, which is common and associated with colony losses. In this issue, Nazzi and Pennacchio provide a focused review and perspective on the role of NF-κB mediated antiviral responses in controlling DWV infections in honey bees. This perspective nicely illustrates the influence of additional abiotic and biotic factors, including the synergistic negative impacts of an ectoparasitic mite that vectors DWV, the insecticide clothianidin, and nutritional stress [7].

The most well-studied invertebrate antiviral response are those of Dipteran insects, specifically, *Drosophila melanogaster* and mosquitoes of the genera *Aedes* and *Culex*. In this issue, Palmer, Varghese, and van Rij provide a thorough and extensive review of antiviral innate immune mechanisms and the variation in resistance to virus infection in co-evolved insect host—virus pairs [2]. This synthesis is an important contribution to the field; it includes information on over 28 viruses and several dipteran hosts, including the evolution and identification of virus resistance loci [2]. Furthermore, the impact of other microbes, sexual dimorphism, infection history, and the role of endogenous viral elements and integrated non-retroviral RNA virus sequences, on the outcome of virus infections are discussed [2]. Swevers, Liu, and Smagghe also review antiviral defense in *Drosophila*—with an emphasis on better understanding the relative importance and involvement of RNAi mechanisms (i.e., siRNA, piRNA, miRNA pathways), as compared to other innate immune pathways, virus-specific antiviral and pro-viral host-encoded genes, and the heat shock response [3]. Whereas, Monsanto-Hearne and Johnson focus on the contribution of *Drosophila* miRNAs in antiviral defense against arboviruses [8].

Invertebrates are rarely challenged with a single pathogen at any given time, therefore considering the impact of additional microbes on the outcome of virus infection is critical [2,3,5]. To this end, Hardy and colleagues describe the virus-reducing impact of *Wolbachia*, a maternally transmitted endosymbiotic bacterium, in mosquitoes, which would in turn lessen the number of mosquito to human transmission events [5]. For example, *Wolbachia* strains *w*Mel and *w*AlbB limit mosquito reproduction and inhibit virus replication in mosquitoes (reviewed in [5]). Though there have been successful pilot programs that utilized *Wolbachia* for mosquito population control, the molecular mechanisms of the bacteria—virus—host relationship are not understood, therefore Hardy and colleagues review Wolbachia-host and RNA virus-host interactions at the cellular level and clearly illustrate overlapping responses, particularly cellular stress responses that would impact the outcome of both infections and may result in restricting virus infections [5].

Original research articles provide a glimpse into cutting-edge research in invertebrate immunology. Specifically, Adelman and Myles expand our understanding of the repertoire and functions, including proviral and antiviral roles, of C-type lectin domain containing proteins (CTLDcps) in *Aedes aegypti* mosquitoes, and update the nomenclature used to describe proteins in this family [9]; Wang, Gu, and colleagues review the role of piRNAs in mosquito antiviral responses and characterize the small RNA profiles of dengue-infected adult female *Ae. albopictus* mosquitoes [10]; Traub-Cseko, Tempone, and colleagues identify proteins involved in the non-sequence-specific dsRNA antiviral response mounted by sand flies (*Lutzomyia longipalpis*) [6]; van Oers, Ros, and colleagues describe a baculovirus (Spodoptera exigua multiple Nucleopolyhedrovirus) encoded protein tyrosine phosphatase 2 that induces mild apoptosis in *Spodoptera frugiperda* (Sf) 21 cells [11]; and Martin, Schroeder, and colleagues describe the development and utility of a variant-specific quantitative RT-PCR assay required to further investigate the potential differential distribution, abundance, and pathogenesis of bee-infecting deformed wing virus master variants A, B, and C [12].

Together the articles in this issue serve as a strong foundation and inspiration for future studies aimed at elucidating the molecular mechanism of invertebrate antiviral defense.
